# Research and Remedies

**Published:** 1912-06-15

**Authors:** 


### RESEARCH AND REMEDIES.

### The Clinical Estimation of Blood Pressure.

In the *Dublin Journal of Medical Science* Dr. Nesbitt comments adversely on the many instruments which are in use for ascertaining the systolic and diastolic arterial blood pressure. He fully agrees that these estimations are capable of affording great help in diagnosis and treatment, but maintains that in practice clinically they are often incorrect. He considers in turn the various types of sphygmomanometer and hæmomanometer on the market; on the whole he prefers the "armlet" class, in which obliteration of the pulse wave is brought about by air pressure in a distensible rubber bag applied to the whole circumference of the arm. Even with the best of these there are numerous sources of error. 1. The pressure needed to flatten the arterial wall itself, independent of the blood tension within it, is unknown and probably variable within wide limits. 2. Different instruments give different readings on the same patient; and this even happens when they are of identical pattern and manufacture. 3. The degree of tightness with which the armlet is adjusted affects the result; the looser the band is applied the higher the readings. 4. The width of the armlet also has some influence: the narrower the band the higher will the readings be. Differences up to 60 mm. have been recorded. A width of 12 cm. at least is regarded as essential. 5. The elasticity of the limb tissues may vary and so prejudice the observed pressures. It is suggested that this factor may vary as much as 30 to 50 mm. 6. Aneroid scales are condemned as liable to get out of order quickly, especially if subjected to excessive or sudden pressures. 7. Spirit indexes are equally unreliable owing to their response to changes of temperature. 8. All armlet methods are open to the objection that compression of the limb may of itself raise the general blood pressure. Gartner's tonometer avoids this factor but introduces others just as disturbing. Then, besides, there are fallacies in the estimation of diastolic pressure. The patient himself is also responsible for disturbances. Age, sex, body weight, atmospheric pressure, digestion, posture, muscular exertion, emotion, and other common causes all have an influence. The author concludes by pleading for the cultivation of the digital perceptions.

### Intravenous Injections.

So numerous are the substances which have been and are given by intravenous injection that the intricacies of technique are gradually being worked out. Salvarsan, ether, hedonal, and many other drugs, to say nothing of the long familiar normal saline solution, are being administered on a grand scale; and, with increasing experience, the pyrexial complications of some of these therapeutic methods have forced themselves into notice. At the moment, the view would seem to be that such deviations from the normal are due, not to the drug injected, but to bacterial contamination of the solution used. Wechselmann claims to have established this in connection with salvarsan, and some of the authorities in this country believe that he is right. Even normal saline has been occasionally found to produce effects the reverse of pleasant. Hort and Penfold adopt the same explanation as that of Wechselmann with respect to salvarsan—namely, that the saline solution has not on such occasions been perfectly sterile. It would appear from the researches of bacteriologists that saline solutions, so often given alone or with other drugs dissolved, are very difficult to preserve sterilised. The moral drawn is that such solutions for intravenous use should be freshly made with freshly distilled water and should be sterilised in an autoclave. It has been shown that distilled water may contain bacilli microscopically indistinguishable from the *Bacillus tuberculosis*. These refinements of technique will not make the new methods any more generally useful; for they mean that their exponents will be more than ever the specialists, and not the general practitioners.

